# Oncolytic virus VT1092M synergizes with immune checkpoint inhibitors against intraperitoneal metastases of colorectal cancer

**DOI:** 10.3389/fimmu.2026.1926207

**Published:** 2026-09-30

**Authors:** Zongfeng Hu, Xinru Yuan, Shuzhen Li, Wenqi Liu, Jiajia Liu, Hua Zhou, Chunyang Sun, Chao Tian, Kaixuan Xue, Yiting Cheng, Jinyu Wang, Haitian Xu, Jianshuai Yang, Xiaopeng Li, Jingfeng Li

**Affiliations:** 1 School of Pharmacy, Yantai University, Yantai, Shandong, China; 2 Beijing WellGene Company, Ltd, Beijing, China

**Keywords:** colon cancer, HSV-1, ICIS, intraperitoneal metastasis, oncolytic virus

## Abstract

**Background:**

Intraperitoneal metastasis is a frequent progression in colon cancer, highlighting the critical need for enhanced therapeutic approaches. Oncolytic virus (OV) therapy has emerged as a promising immunotherapeutic strategy. The combination of OVs with immune checkpoint inhibitors (ICIs) has demonstrated an enhanced anti-tumor response.

**Methods:**

We generated the intraperitoneal-subcutaneous bilateral colon cancer xenograft model to simulate intraperitoneal metastatic colon cancer, and evaluated the systemic antitumor efficacy of the oncolytic HSV-1 virus VT1092M in combination with ICIs (anti-PD-L1 antibody and anti-CTLA-4 antibody).

**Results and discussion:**

Intraperitoneal administration of VT1092M markedly suppressed intraperitoneal cancer progression and enhanced the superior antitumor efficacy of ICIs immunotherapy in murine intraperitoneal metastatic colon cancer. Notably, treatment of VT1092M combined with anti-CTLA-4 antibody could eliminate both intraperitoneal metastases and subcutaneous tumors, while also inhibiting the formation of peritoneal blood vessels and malignant ascites. The synergistic combination of VT1092M and anti-CTLA-4 elicited potent intraperitoneal and systemic antitumor immunity, culminating in durable, tumor-specific memory that significantly prolonged survival in mice with colorectal intraperitoneal metastases compared to either monotherapy. Mechanistically, the combination therapy activates the anti-tumor immune environment within the peritoneal cavity, leading to a significant increase in tumoricidal CD8^+^ T cells and M1 macrophages, as well as the reprogramming of transcriptional signatures associated with the tumor immune response within the tumor microenvironment. Ultimately, intraperitoneal immunotherapy with VT1092M enhances the efficacy of immune checkpoint blockade in suppressing intraperitoneal metastatic colorectal cancer and induces durable tumor-specific immune memory, thereby exerting substantial anti-tumor effects. This finding highlights the potential of synergistically combining oncolytic viruses with ICIs to advance immunotherapeutic strategies for intraperitoneal metastatic colorectal cancer.

## 1 Background

Colon cancer (CC) is a prevalent gastrointestinal malignancy and remains a significant global health concern, ranking among the leading causes of cancer-related mortality (1–3). By 2030, the global prevalence of CC is anticipated to increase by 60%, resulting in over 2.2 million new cases and more than 1.1 million deaths (4). Patients with metastatic colorectal cancer face a particularly poor prognosis, with a 5-year survival rate of less than 15% (5, 6). Peritoneal metastasis represents a common progression pattern that complicates traditional treatment approaches, necessitating the urgent development of novel therapeutic strategies (7, 8).

Immune checkpoint inhibitors (ICIs) have shown substantial efficacy in the treatment of various cancers, particularly in certain refractory solid tumors (9, 10). However, not all tumor types demonstrate a favorable response to ICIs as a monotherapy; likewise, within CC, not all subtypes exhibit a positive response, especially advanced malignant tumors with intraperitoneal metastasis (11–16). This limited effectiveness is attributed to the complexity of the tumor microenvironment, immune escape mechanisms, and the paucity of tumor-infiltrating lymphocytes (TILs) (17, 18).

Oncolytic virus (OV) therapy is a promising cancer immunotherapy that selectively infects and destroys tumor cells, triggering an immune response by releasing tumor antigens, which are presented by dendritic cells (DCs), thereby activating an anti-tumor immune response (19, 20). As a result, OV acts as a local cancer vaccine within the tumor microenvironment (TME). This makes OV an attractive therapeutic approach, capable of enhancing cytotoxic effector cells and inducing an antigen-specific T-cell response. Studies have shown that OVs can enhance the efficacy of ICIs by remodeling the tumor microenvironment (21, 22). The combination of OV therapy and ICIs not only restores peritoneal anticancer immunity but also boosts anti-tumor immune responses through various mechanisms, ultimately achieving effective control of peritoneal metastatic CC (23, 24).

The OV VT1092M, derived from the oncolytic HSV-1 virus VT09X with deletions in the γ34.5 and α47 genes and expressing murine IL-12, has exhibited significant anti-tumor efficacy in subcutaneous cancer models (25–27). Two of our oncolytic candidates—VT1092 (encoding human IL-12, CTR20220801) and VT1093 (encoding human anti-PD-1 antibody, CTR20210659)—have been completed phase I clinical investigation for safety and efficacy in advanced solid tumors. However, subcutaneous models do not accurately replicate the distinct immunosuppressive and angiogenic characteristics of peritoneal carcinomatosis, such as the formation of malignant ascites and vascular leakage. The therapeutic potential of intraperitoneal VT1092M, relevant for peritoneal metastases, and its synergy with ICI blockades remain unexplored. Here, we aimed to evaluate the therapeutic efficacy of intraperitoneal injection with VT1092M in intraperitoneal metastatic CC, focusing on its potential in combination with ICIs to induce systemic antitumor responses. Our study shows that the OV VT1092M, when combined with ICIs, particularly the anti-CTLA-4 antibody, effectively suppresses intraperitoneal metastatic CC growth and exhibits a more potent systemic anti-tumor response. These findings offer a theoretical foundation and promising prospects for the clinical application of OV in conjunction with ICIs for the treatment of metastatic CC.

## 2 Materials and methods

### 2.1 Cell lines

The primary mouse CC cell line CT26.WT and the mouse breast cancer 4T-1 cell lines were obtained from the National Collection of Authenticated Cell Cultures, while the mouse CC CT26 cell line stably expressing luciferase (CT26-luc) came from the Shanghai Model Organisms Center, Inc. All three were cultured in RPMI 1640 medium with 10% FBS (Gibco, Logan, Utah, USA) and 1% penicillin–streptomycin (Invitrogen) at 37 °C in a 5% CO_2_ chamber. The Vero cell line was sourced from ATCC and cultured in DMEM with 10% FBS (Gibco, USA) under similar conditions for virus amplification.

### 2.2 Viruses

All the viruses used in this study were supplied by Beijing WellGene Biotechnology Co., Ltd. (Beijing, China). The control virus, VT09X, was derived from the wild strain HL-1 isolated from an Asian patient infected with HSV-1, with the neurotoxic ICP34.5 and MHC-I inhibiting ICP47 genes removed (28). The OV, VT1092M, previously reported, includes the murine *IL-12* gene inserted into VT09X (25). Both viruses were replicated in Vero cells, stored at −80 °C, and purified following established methods (22). The recombinant virus titers used in this study were 2 × 10^8^ PFU/ml.

### 2.3 *In-vivo* experiments and tumor models

All animal experiments adhered to the Guidelines for the Care and Use of Laboratory Animals and were approved by the Animal Care and Use Committee of Yantai University (IACUC No. 2018-DA-12). BALB/c mice (5–6 weeks old) were obtained from Jinan Pengyue Laboratory Animal Breeding Co. Ltd. (Animal License No.: SCXK (lu) 2022–0006) and were maintained in a specific pathogen-free environment at the animal facility of Yantai University, China. In the viral intraperitoneal tolerance dosing assay, mice were randomly assigned to four groups (*n* = 6/group) and administered intraperitoneal injections of either 250 μl sodium chloride (NaCl) or VT1092M at doses of 2.5 × 10^7^ PFU, 5 × 10^7^ PFU, or 1 × 10^8^ PFU in a volume of 250 μl on Days 1, 3, 5, 7, 9, and 11. Daily body weight measurements were recorded, and observations were made regarding piloerection, feeding, drinking, excretion, and general activity. Additionally, survival was monitored over a 70-day period.

Studies showed that luciferase expression and bioluminescence do not affect tumor growth, validating their use in tumor models (29). For the efficacy of the VT1092M experiment, BALB/c mice (5–6 weeks old) were injected intraperitoneally with 5 × 10^5^ CT26-luciferase cells to simulate colorectal cancer spread and subcutaneously with 1 × 10^6^ CT26.WT cells on the right dorsal side to create a bilateral tumor model. Mice with intraperitoneal tumors exhibiting a total flux of 1 × 10^10^ to 5 × 10^10^ photons/sec were randomly assigned to three groups (*n* = 6), and the day of grouping was considered as the first day. Mice were respectively received intraperitoneal injections of 250 μl NaCl, 1 × 10^7^ PFU VT09X, and 1 × 10^7^ PFU of VT1092M in a volume of 250 μl at Days 1, 3, 5, 7, 9, and 11. Every two days, *in-vivo* imaging assessed intraperitoneal tumor luminescence, while subcutaneous tumor volume, waist circumference, and body weight were recorded. The increase in subcutaneous tumor volume percentage indicated tumor growth. Each group of mice underwent a 70-day survival monitoring, ending upon natural death or when the tumor exceeded 2,000 mm³.

For evaluation of systemic antitumor effects of VT1092M combined with ICIs on a mouse colorectal intraperitoneal metastatic model, the mice were then randomly allocated into seven groups based on the total flux of intraperitoneal tumors, ranging from 1 × 10^10^ to 5 × 10^10^ photons per second. Mice were respectively treated with control NaCl and control VT09X virus, the VT1092M virus (1 × 10^7^ pfu/mouse, i.p.) 6 times on Days 1, 3, 5, 7, 9, and 11, as well as control anti-PD-L1 (i.p., 200 μg/mouse, Bioxcell, Cat No. BP0101, USA) on Days 1, 5, and 8 or anti-CTLA-4 antibody (i.p., 300 μg/mouse, Bioxcell, Cat No. BP0164, USA) on Days 1 and 8. For combination therapy, each mouse received six intraperitoneal injections of the VT1092M virus (1 × 10^7^ pfu/mouse) on Days 1, 3, 5, 7, 9, and 11, in conjunction with either anti-PD-L1 (200 μg/mouse, Bioxcell, USA) on Days 1, 5, and 8, or anti-CTLA-4 antibody (300 μg/mouse, Bioxcell, USA) on Days 1 and 8 starting 1 week after tumor inoculation (**Figure 1A**). *In-vivo* imaging was employed to evaluate intraperitoneal tumor luminescence, while subcutaneous tumor volume and body weight were recorded bi-daily. The subcutaneous tumors were measured with a caliper and calculated using the standard formula: (length × width^2^) × 0.5. The increase in the percentage of subcutaneous tumor volume was indicative of tumor progression. Following treatment, the mice were euthanized by isoflurane inhalation (RWD; Cat. No. R-510-2216; 1% inhalation, 0.5 L/min) followed by cervical dislocation, and tissues were collected for subsequent analysis. Seven mice from each group were subjected to a 70-day survival monitoring period and ended upon natural death or when the subcutaneous tumor exceeded 2,000 mm³ to evaluate the survival outcomes associated with each treatment group. All procedures incorporated blinding and randomization across animal handling, dosing, and outcome assessments.

**Figure 1:**
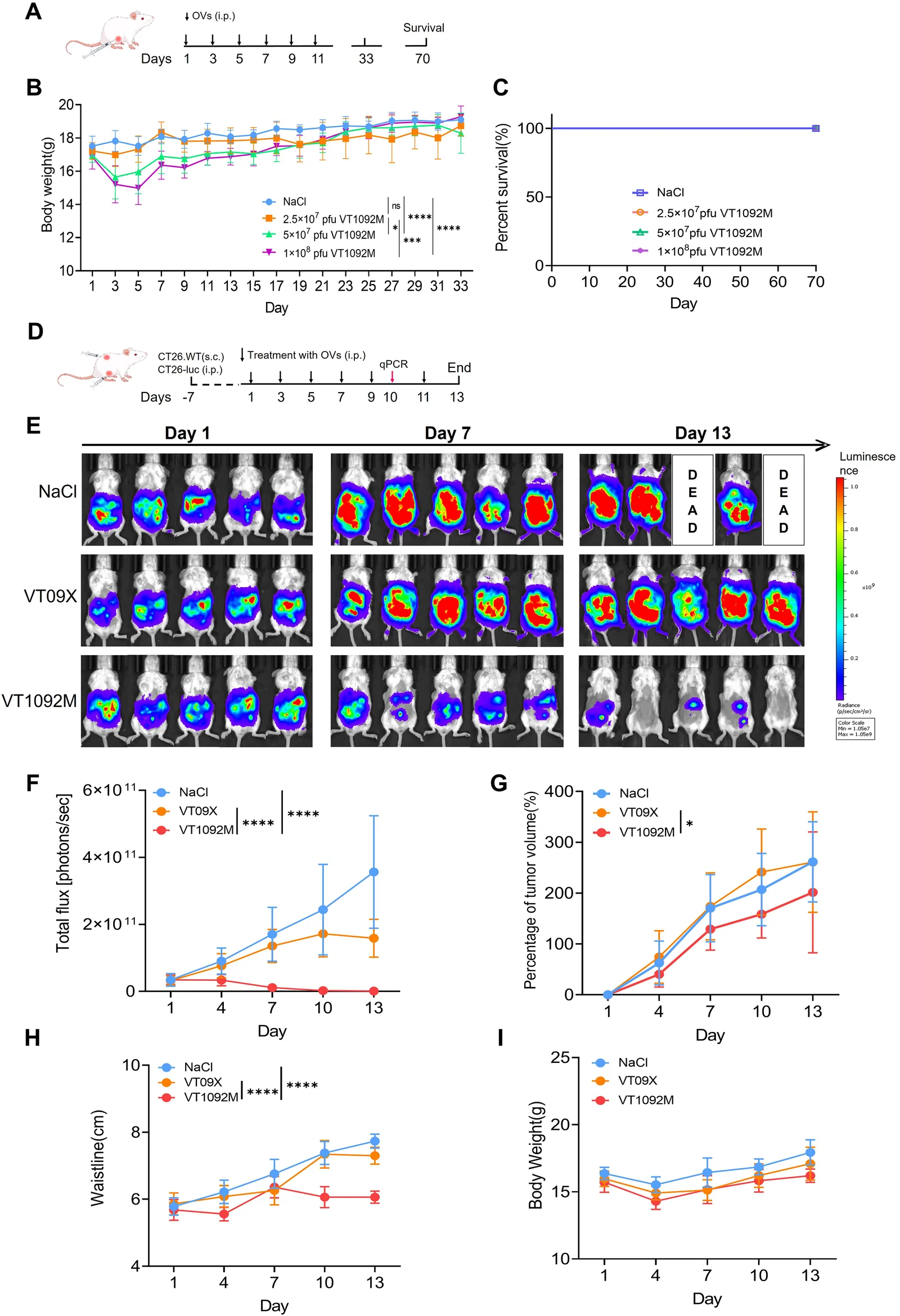
Effect of VT1092M on intraperitoneal-subcutaneous bilateral tumors. **(A)** BALB/c mice (*n* = 6/group) received intraperitoneal injections of 250 μl NaCl or 2.5 × 10^7^ PFU, 5 × 10^7^ PFU, 1 × 10^8^ PFU of VT1092M in a volume of 250 μl at Days 1, 3, 5, 7, 9, and 11. **(B)** Body weight curves and **(C)** the survival time of mice were recorded for 70 days. **(D)** The anti-tumor effect of VT1092M on intraperitoneal-subcutaneous bilateral colon cancer xenografts. Mice were randomly allocated into three groups and respectively received intraperitoneal injections of 250 μl NaCl, 1 × 10^8^ PFU of VT1092M or VT09X at Days 1, 3, 5, 7, 9, and 11 starting 1 week after tumor inoculation. (**E, F**) Fuminescence changes in the intraperitoneal tumor in mice were measured. **(G)** Subcutaneous tumor volume in mice was measured to calculate the percentage increase. **(H)** Waistline curves and **(I)** Body weight curves of mice in VT1092M treatment (*n* = 6). The experimental results are expressed as Mean ± SD, *P < 0.05, ***P < 0.001, ****P < 0.0001.

### 2.4 Bioluminescence imaging

Intraperitoneal tumor progression was assessed through luciferase expression in infected mice utilizing the *in-vivo* imaging system (IVIS) Lumina Series III (PerkinElmer LAS, Germany). Prior to imaging, the mice were shaved to reduce light absorption by the fur. Briefly, the mice were anesthetized with 2% isoflurane (1% inhalation, 0.5 L/min) and administered an intraperitoneal injection of 100 µl of 30 mg/ml luciferase substrate, Xenolight™ D-Luciferin Potassium Salt (Cat. No. 122799, PerkinElmer, USA). Photon emission was subsequently measured 5 min post-injection using the *in-vivo* imaging system, with an acquisition time for intraperitoneal tumor-bearing mice. Image analysis was conducted using Living Image software (Xenogen) and obtaining maximum values in photons/second/cm^2^/steradian.

### 2.5 qPCR analysis

To assess the biodistribution of the OV in mice following intraperitoneal administration, quantitative PCR (qPCR) for absolute quantification was performed using the Roche LightCycler 480 PCR System (Germany). Tumor tissues from both intraperitoneal and subcutaneous models, along with spleen, liver, and blood samples, were collected from the mouse cohorts after five treatment doses. Genomic DNA was extracted utilizing the DNeasy Blood and Tissue Kit (Cat. No. 69506, Qiagen, Germany). The qPCR assays were conducted with the Probe qPCR Mix (Cat. No. D7518, Beyotime) and specific primers. The absolute viral concentration was determined based on the equation derived from the standard curve and subsequently converted into copy numbers per microgram of genomic DNA. The probes and primers employed in this study are detailed in **Supplementary Table S1**.

### 2.6 Flow cytometry analysis

The flow cytometry analyses of the intraperitoneal tumors, ascites and peripheral blood in mice were employed by a multicolor FACSCelesta Flow Cytometer (BD Biosciences, San Jose, CA) to study different immune cell subsets. All the antibodies used in the flow cytometry assay were sourced from BioLegend (San Diego, CA, USA). On the 10th day of treatment, the intraperitoneal tumors and ascites were harvested from each group of mice. For the VT1092M and combination groups with minimal ascites, the peritoneal cavity was washed with saline to collect cells. Tumor single-cell suspensions were prepared using the Tumor Dissociation Kit (Miltenyi Biotec, Gladbach, Germany) with the gentleMACS™ Octo Dissociator with Heaters (Miltenyi Biotec), and ascites were lysed with a red blood cell lysis buffer (Cat. No. 103094183, Miltenyi, Germany). On the 14th day of treatment, peripheral blood was collected from each group of mice using the retro-orbital bleeding method following ethical guidelines and transferred into Eppendorf tubes containing 100 μl of heparin sodium solution (28). Red blood cells were lysed with a red blood cell lysis buffer. The resulting single-cell suspensions from mice were equally aliquoted into two flow cytometry tubes. Each aliquot was pre-treated with 1 μl anti-mouse CD16/32 antibody (TruStain FcX™ PLUS, Cat No. 156603) to block non-specific antibody for 5 min at 4 °C in the dark, followed by PBS washing and Zombie Aqua™ Fixable Viability Kit staining (Cat No. 423101, BioLegend) according to the manufacturer’s protocol to identify dead cells. For immunophenotyping, the following antibody panels were utilized: Tube 1 involved cell surface staining with CD45-AF700 (Cat No. 103140), CD3-PerCP-Cy5.5 (Cat No. 100328), CD4-APC-Cy7 (Cat No. 100414), CD8-FITC (Cat No. 100706), CD44-BV421 (Cat No. 103040), and CD62L-PE (Cat No. 104407). Tube 2 included surface staining with CD45-AF700 (Cat No. 103140), CD3-PerCP-Cy5.5 (Cat No. 100328), CD335-BV421 (Cat No. 137612), and B220-FITC (Cat No. 103206). The stained samples were incubated for 30 min at room temperature (light-protected), washed twice with PBS, and then analyzed using a flow cytometer (FACSCelesta, BD Biosciences) with standardized voltage and compensation settings. Data obtained were examined using FlowJo version 10 (BD Biosciences).

### 2.7 Multicolor immunofluorescence staining assay

The intraperitoneal tumors were harvested from mice in the NaCl, anti-CTLA-4, and VT1092M+anti-CTLA-4 groups on Day 10 of treatment. Multicolor immunofluorescence staining (mIF) assays were conducted following the Univ-Bio Company (Shanghai, China) protocols. The tumor tissues underwent fixation in ethanol, embedding, and sectioning, succeeded by antigen retrieval and incubation with a 3% H_2_O_2_ methanol solution at ambient temperature. Subsequently, the sections were washed thrice with PBST. A blocking solution was applied to prevent nonspecific antibody binding. The tissue sections were then incubated with primary antibodies HSV-1 ICP8 (sc-53329, Santa Cruz Biotechnology); CD4 (ab183685, Abcam); CD8 (ab217344, Abcam); CD68 (Boster, BA3638); iNOS (ab15323, Abcam); CD206 (#24595, Cell Signaling Technologies); and corresponding secondary antibodies conjugated with fluorescent dyes. Following antibody elution, nuclei were counterstained with 4′,6-diamidino-2-phenylindole (DAPI). Image acquisition and analysis were performed using the 3D Histech Pannoramic MIDI (3D HISTECH Ltd., Budapest, Övu.3., Hungary) and AKOYA Vectra Polaris systems. Statistical analyses were carried out using HALO Image Analysis Software (Indica Labs).

### 2.8 ELISA assay

Intraperitoneal tumors were obtained from the NaCl, anti-CTLA-4, and VT1092M+Anti-CTLA-4 groups following 10 days of treatment. The tumor tissues were weighed and homogenized, and subsequent centrifugation at 12,000 × g for 10 min allowed for the collection of supernatants for ELISA analysis. Levels of IFN-γ and IL12 were quantified using ELISA kits specific for mouse IFN-γ (SEKM-0031) and IL12 (SEKM-0013), both procured from Solarbio. Data analysis was conducted using GraphPad Prism 9.0 software, and protein concentrations were reported relative to tissue weight (pg/mg).

### 2.9 Cytotoxicity assay

The peripheral blood was collected from mice assigned to the NaCl, VT09X, VT1092M, anti-CTLA-4, and VT1092M+anti-CTLA-4 groups 2 days after treatment ends. Erythrocytes were lysed using a red blood cell lysis buffer, followed by two washes with 1 × PBS. Non-viable cells were eliminated using the Dead Cell Removal Kit (Cat. No. 130090101, Miltenyi). CD45^+^ cells were isolated employing CD45 (TIL) Microbeads (Cat. No.130052301, Miltenyi) in conjunction with an LS separation column (Cat. No. 130042401, Miltenyi) and a QuadroMACS™ Separator (Cat. No. 130090976, Miltenyi). Target cells, specifically CT26.WT and 4T1, were labeled with carboxyfluorescein succinimidyl ester (CFSE) at 37 °C for 20 min, followed by two washes with the cold RPMI 1640 medium. The cells were quantified using the Countess 3 automated cell counter (Thermo Fisher Scientific). The stained target cells and effector cells were combined in a 24-well plate and incubated at 37 °C with 5% CO_2_ for 4h according to the specified target-to-effector ratio (E:T). Subsequently, the co-incubated cells were then stained with propidium iodide (PI), and the cytotoxic efficacy of CD45^+^ cells was assessed using flow cytometry. The killing efficiency was determined using the formula: Killing Efficiency = (CFSE^+^/PI^+^)/(CFSE^+^/PI^+^ + CFSE^+^/PI^-^) × 100%.

### 2.10 RNA sequencing and analysis

Intraperitoneal and subcutaneous tumor samples from BALB/c mice in NaCl, VT09X, VT1092M, anti-CTLA-4, and VT1092M+anti-CTLA-4 groups were collected 2 days after the final treatment. The small tumor size in the combination group necessitated the pooling of mice for sequencing. These samples were sent to Azenta Lifesciences for RNA sequencing. The resultant RNA quality and quantity were assessed and subsequently utilized for library preparation. Sequencing was conducted on an Illumina NovaSeq MGI2000 platform at Azenta Lifesciences. Base calling was performed using the Bcl2fastq software (version 2.20.0.422), generating raw data, which was then filtered using Cutadapt (version 1.9.1). The clean reads were mapped to the Mus musculus genome (NCBI build GRCm38) using HISAT2 (version 2.2.1) (30). Read quantification was carried out with HTSeq (version 0.6.1), and expression values were normalized to fragments per kilobase of exon per million fragments mapped (FPKM). Hierarchical clustering was conducted on the log_10_-transformed (FPKM + 1) values of the union of differentially expressed genes across all comparison groups. To identify pathways enriched in VT1092M+anti-CTLA-4–treated tumors, Gene Set Enrichment Analysis (GSEA) was performed using the pre-ranked option, with genes ranked according to log2-fold changes between each group. Gene Ontology (GO) enrichment analysis was executed using GOseq (v1.34.1) based on the Wallenius non-central hypergeometric distribution. KEGG pathway enrichment was similarly examined using the hypergeometric test.

### 2.11 Re-challenge experiment

Following the 55-day survival period of the *in-vivo* efficacy study, three mice from the VT1092M+anti-CTLA-4 treatment group, which had been cured and exhibited no tumor recurrence, were randomly selected for a tumor re-challenge experiment. Additionally, three age-matched control BALB/c mice were selected. On Day 56, CT26-Luc cells and CT26.WT cells, in quantities identical to those used for initial tumor induction, were re-inoculated into the peritoneum and subcutaneously, respectively. Beginning on Day 61, the mice were monitored every two days for body weight, subcutaneous tumor volume, and overall condition. Bioluminescence changes in intraperitoneal tumors were assessed using an *in-vivo* imaging system (IVIS) Lumina Series III to determine whether the VT1092M+Anti-CTLA-4 treated mice, after achieving remission, possess the capacity to resist re-challenge by the same tumor cells.

### 2.12 Date analysis

Data are shown as mean ± SD, and statistical significance was determined using one-way ANOVA and Dunnett’s *post hoc* test. *P*-values of < 0.05 were considered statistically significant. Kaplan–Meier survival analysis was used for overall survival studies, and statistical significance was calculated using the Mantel-Cox test.

## 3 Results

### 3.1 The anti-tumor effect of VT1092M on intraperitoneal-subcutaneous bilateral CC xenografts

Peritoneal metastasis poses a significant clinical challenge in colorectal cancer, leading to a poor prognosis and restricted therapeutic options. Although our prior research established the anti-tumor efficacy of VT1092M in subcutaneous CC models (25, 26), its therapeutic potential against intraperitoneal metastasis—a clinically more aggressive and anatomically intricate tumor environment—has not yet been investigated. We therefore sought to evaluate the efficacy of VT1092M against intraperitoneal metastatic CC and its potential to induce systemic antitumor immune responses. As a first step, we determined the safe dosing regimen for intraperitoneal injection of VT1092M. Mice treated with VT1092M exhibited varying piloerection, most severe in the 1×10^8^) pfu and 5 × 10^7^ pfu groups on Day 2, subsiding by Day 7. Significant weight loss occurred from Days 1 to 3 in the 5 × 10^7^ pfu and 1 × 10^8^ pfu groups compared to the NaCl group (*P* < 0.0001). By Day 5, the 1 × 10^8^ pfu group continued losing weight, while the 5 × 10^7^ pfu group began gaining weight. By Day 7, the 1 × 10^8^ pfu group started recovering weight, and the 2.5 × 10^7^ pfu group did not experience significant weight loss. No mortality occurred during the 70-day observation, indicating that 2.5 × 10^7^ pfu VT1092M for intraperitoneal injection was well tolerated (**Figures 1A–C**).

To assess the inhibitory effect of VT1092M on intraperitoneal metastatic CC and its potential to elicit systemic antitumor responses, we examined the anti-tumor activity of VT1092M on intraperitoneal-subcutaneous bilateral CC xenografts in BALB/c mice (**Figure 1D**). Mice were randomly allocated into three groups (*n* = 6/group) and respectively received intraperitoneal injections of 250 μl NaCl, 1 × 10^8^ PFU of VT1092M, or VT09X at Days 1, 3, 5, 7, 9, and 11, starting 1 week after tumor inoculation. As expected, VT1092M-treated mice exhibited marked reductions in intraperitoneal tumor burden, demonstrating superior efficacy compared to both controls and VT09X monotherapy (**Figures 1E, F**). In contrast, subcutaneous tumor growth rates showed a significant difference between the VT1092M and VT09X groups, while there was no significant reduction in the control group (**Figure 1G**). Following five intraperitoneal injections of OVs, we analyzed their distribution within intraperitoneal and subcutaneous tumors, as well as in major organs. The viral load was predominantly concentrated in the intraperitoneal tumors, whereas no viral load was detected in subcutaneous tumors, blood, or in the spleen and liver (**Supplementary Figure S1**). The findings elucidate a compartment-specific antitumor activity profile for VT1092M, marked by strong local suppression of intraperitoneal malignancies, yet exhibiting limited systemic immune activation for the control of distal subcutaneous lesions.

### 3.2 VT1092M improves the efficacy of ICI therapy for intraperitoneal colorectal metastases

Next, we assessed the efficacy of VT1092M in combination with either anti-PD-L1 or anti-CTLA-4 antibodies in the intraperitoneal-subcutaneous bilateral CC xenograft model. Mice were i.p. injected with VT1092M, VT09X, or NaCl (as a control) 6 times (Days 1, 3, 5, 7, 9, and 11) at a dose of 1 × 10^7^ pfu and were treated with an anti-PD-L1 antibody (Days 1, 5, and 8) or an anti-CTLA-4 antibody (Days 1 and 8) starting 1 week after tumor inoculation (**Figure 2A**). As expected, the combination groups (VT1092M+Anti-CTLA-4, VT1092M+Anti-PD-L1) and the VT1092 group sustained a reduction in abdominal tumor burden as early as Day 7 after treatment (**Figures 2B, C**). Furthermore, the combination groups exhibited significantly enhanced efficacy in inhibiting abdominal tumor growth compared to both the antibody monotherapy (*P* < 0.0001) and VT1092 groups (*P* < 0.05) after treatment. Notably, the combination groups further significantly decreased subcutaneous tumor growth compared to the respective monotherapy groups (*P* < 0.0001), with the VT1092M+Anti-CTLA-4 group exhibiting the most pronounced inhibitory effect (**Figure 2D**). Additionally, progressive abdominal distension attributable to tumor expansion and hemorrhagic ascites accumulation was observed during treatment. However, mice in the combination group showed significantly smaller waist circumference than those in the monotherapy and control groups (**Figure 2E**). Body weight trends showed marginal increases in all groups without intergroup significance (**Figure 2F**).

**Figure 2:**
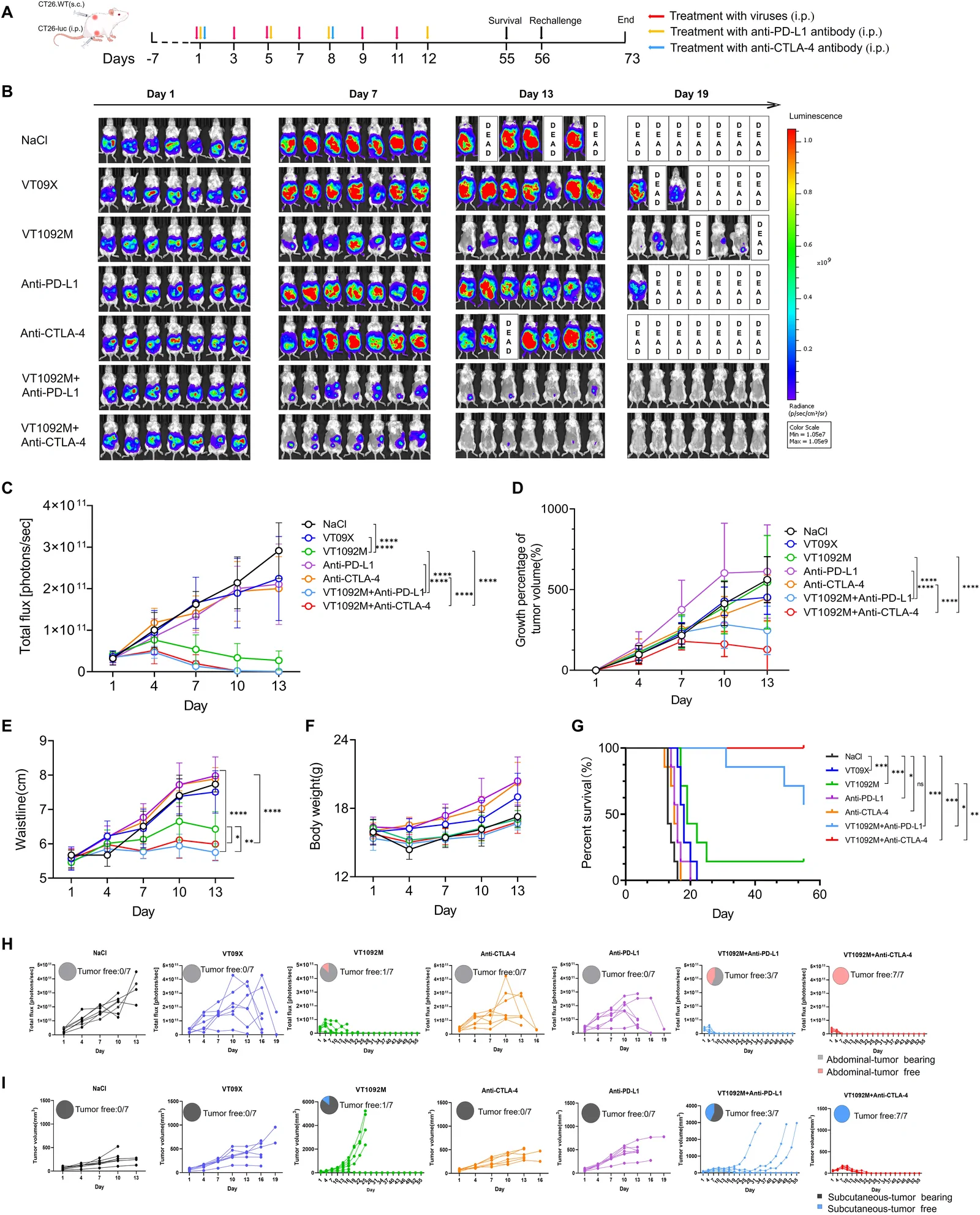
Effect of VT1092M combined with ICIs on bilateral intraperitoneal-subcutaneous colorectal cancer xenografts. **(A)** The timeline shows intraperitoneal treatment with viruses combined with ICIs. Mice were i.p. injected with VT1092M, VT09X, or NaCl (as a control) 6 times (Days 1, 3, 5, 7, 9, and 11) at a dose of 1 × 10^7^ pfu and were treated with anti-PD-L1 (Days 1, 5, and 8) or anti-CTLA-4 (Days 1 and 8) starting 1 week after tumor inoculation. **(B)** Representative bioluminescence images of mouse abdominal tumor in VT1092M combined with ICIs. **(C)** Total photon flux curve of abdominal tumor in VT1092M combined with ICIs. **(D)** Subcutaneous tumor volume growth percentage curves in VT1092M combined with ICIs. **(E)** Body weight curves and **(F)** Waistline curves of mice in VT1092M combined with ICI treatments. **(G)** Effect of VT1092M combined with ICIs on the survival of mice. **(H)** Growth curves of abdominal tumors in each group of mice. **(I)** Growth curves of subcutaneous tumor of in each group of mice (*n* = 7). The experimental results are expressed as Mean ± SD, *P < 0.05, **P < 0.01, ***P < 0.001, ****P < 0.0001.

Then we analyzed the 55-day survival rate and monitored tumor growth in each group. Control group mice exhibited complete mortality by Day 16, while anti-CTLA-4 antibody and VT1092M monotherapy groups reached 100% mortality by Days 17 and 22, respectively. The VT09X group showed full mortality by Day 22. Notably, VT1092M+anti-PD-L1 combination therapy delayed initial mortality until Day 31, achieving a 57.1% survival rate. Strikingly, all mice in the VT1092M+anti-CTLA-4 group survived through the observation period, achieving a 100% survival rate (**Figure 2G**). Longitudinal tumor growth kinetics for individual mice over the 55-day survival period are shown in **Figure 2**. Complete tumor regression without recurrence was observed in abdominal tumors in the VT1092M, VT1092M+Anti-PD-L1, and VT1092M+Anti-CTLA-4 groups, while two mice treated with VT1092M monotherapy succumbed to aggressive abdominal tumor progression by Day 17 (**Figure 2H**). Notably, 4/7 mice in the VT1092M group exhibited progressive subcutaneous tumor regrowth, and 3/7 mice in the VT1092M+Anti-PD-L1 group showed subcutaneous tumor rebound. No recurrence was observed in the VT1092M+Anti-CTLA-4 group (**Figure 2I**). These findings demonstrate that all combination therapies significantly prolonged survival compared to monotherapy. The VT1092M combined anti-CTLA-4 therapy achieved the most robust tumor suppression, maintaining durable remission in both abdominal and subcutaneous tumors in mice.

### 3.3 Combined therapy achieves long-lasting anti-tumor responses

To further evaluate systemic antitumor immunity in cured mice, a tumor re-challenge experiment was conducted using randomly selected 3/6 tumor-free mice from the VT1092M+Anti-CTLA-4 group (**Figure 2A**) and naive controls. Mice received intraperitoneal and subcutaneous injections of CT26-Luc/CT26.WT cells at equivalent doses to initial tumor induction on Day 56 post-primary treatment (**Figure 3A**). Control mice exhibited progressive tumor growth in both intraperitoneal and subcutaneous tumors, whereas combination-treated mice maintained complete tumor-free status and revealed striking therapeutic memory (*P* < 0.0001) (**Figures 3B, C**). These findings demonstrate that VT1092M+anti-CTLA-4 induces durable immunoprotective memory capable of preventing tumor recurrence upon re-challenge, indicative of robust, antigen-specific immune surveillance.

**Figure 3:**
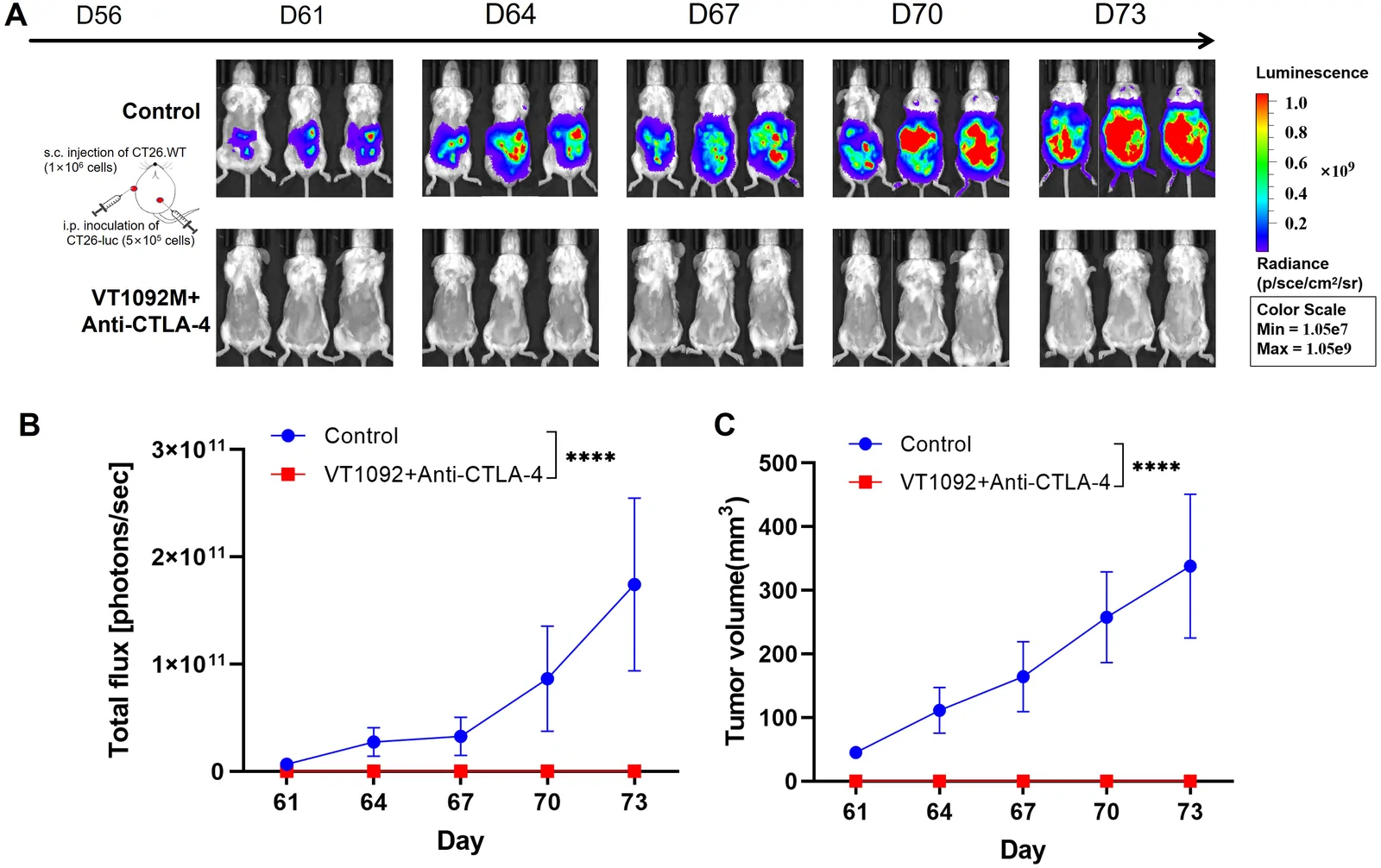
Tumor re-challenge of mice cured by VT1092M combined anti-CTLA-4 antibody. **(A)** Longitudinal monitoring from Day 61 onward biweekly measurements of subcutaneous tumor volume, and bioluminescent flux for intraperitoneal tumors. **(B)** Bioluminescence images of intraperitoneal tumor; **(C)** subcutaneous tumor volume growth curves (*n* = 3). The experimental results are expressed as Mean ± SD, ****P < 0.0001.

### 3.4 VT1092M combined anti-CTLA-4 therapy reprograms the peritoneal immune microenvironment and suppresses angiogenesis

To elucidate the mechanisms underlying the effects of the combination of VT1092M and anti-CTLA-4 antibody on intraperitoneal metastases, we conducted a comprehensive analysis of the peritoneal immune microenvironment during the treatment period. Due to the significant tumor eradication observed in the combination group by the study’s endpoint, we concentrated our analysis on Day 10 of treatment (**Figure 4A**). At both Days 6 and 10 post-treatment, we observed that mice in the NaCl, VT09X, and anti-CTLA-4 antibody groups developed multifocal intraperitoneal tumor metastases on both the visceral and parietal peritoneal surfaces. These surfaces displayed aberrant and malformed neovascularization, as well as persistent accumulation of malignant hemorrhagic ascites, which was accompanied by extensive peritoneal vascular leakage and hemorrhagic foci. In contrast, mice treated with VT1092M, either alone or in combination with anti-CTLA-4 antibody, demonstrated a significantly reduced tumor burden within the peritoneal cavity. Additionally, the volume of ascites in these groups was markedly lower compared to all other monotherapy regimens (**Supplementary Figure S2**).

**Figure 4:**
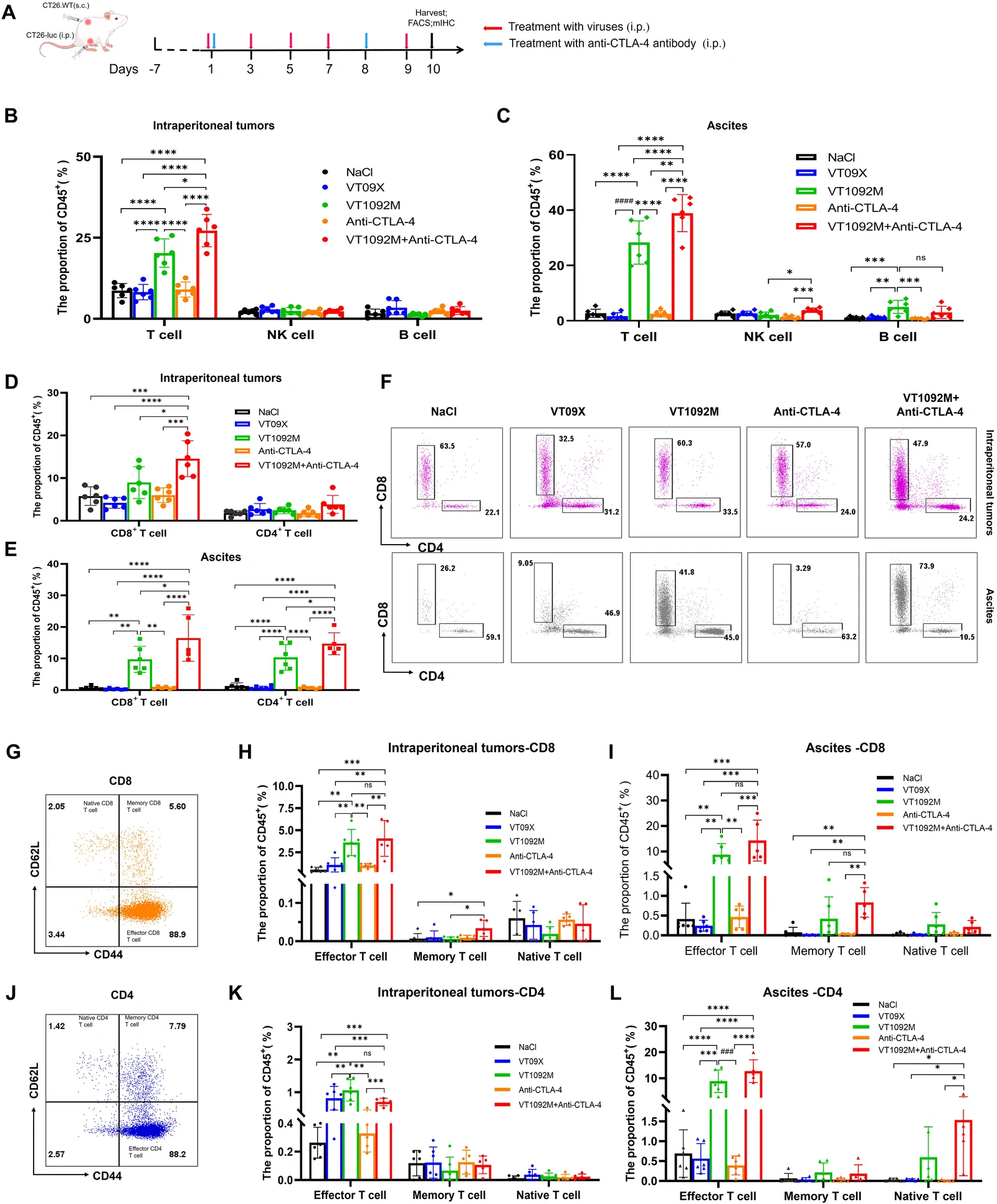
Flow cytometric analysis of immune cell phenotyping on mouse intraperitoneal tumors and ascites after 10 days treatment. The gating strategy of flow cytometric analysis was in Supplementary information S3. **(A)** The intraperitoneal tumors and ascites were harvested from mice in each experimental group and subsequently prepared into single-cell suspensions. The cells were blocked and stained with specific antibodies to analyze T cells and their subsets, as well as NK and B cells. (**B, C**) The proportions of T cells, NK cells, and B cells within the intraperitoneal tumors and ascites; (**D, E**) the proportions of CD8^+^and CD4^+^T cells in the intraperitoneal tumors and ascites; **(F)** representative flow cytometry plots of CD8^+^and CD4^+^T cells in the intraperitoneal tumors and ascites; **(G)** representative flow cytometry plots and proportions of CD8^+^T-cell subsets in the intraperitoneal tumors **(H)** and ascites (**I, J)** representative flow cytometry plots and proportion plots of CD4^+^T-cell subsets in the intraperitoneal tumors **(K)** and ascites (**L**). *n* = 5 to 6 per group, *p < 0.05, **p < 0.01, ***p < 0.001, ****p < 0.0001, ^###^*p* < 0.001, ^####^*p* < 0.0001.

Flow cytometric analysis of intraperitoneal tumors and ascitic cells demonstrated a markedly enhanced anti-tumor immune environment following combination therapy. While the proportions of NK and B cells within the intraperitoneal tumor remained unchanged, combination therapy led to a higher frequency of NK cells in the ascites compared to either monotherapy. Importantly, T-cell infiltration was significantly increased in both ascites and intraperitoneal tumors in the VT1092M and combination therapy groups (**Figures 4B, C**). Subset analysis revealed a notable rise in the percentage of CD8^+^T cells of CD45^+^ cells in both compartments, along with an increase in the percentage of CD4^+^T cells of CD45^+^ cells in ascites within the combination therapy group (**Figures 4D, F**). These infiltrating T cells predominantly exhibited an effector phenotype (CD44^+^CD62L^-^), with a significant enrichment of the percentage of effector CD8^+^and CD4^+^T cells observed in intraperitoneal tumors and ascites (**Figure 4**). Furthermore, the proportions of CD8^+^effector and memory T cells (CD44^+^CD62L^+^) in combination group were significantly increased in both intraperitoneal tumors and ascites compared to the NaCl, VT09X, and anti-CTLA-4 monotherapy groups, indicating the induction of durable anti-tumor immunity (**Figures 4G, I**). Similarly, the proportion of CD4^+^effector T cells in combination group showed a significant increase compared to monotherapy groups, though not statistically significant versus the VT1092M groups. There was also a modest rise in naïve CD4^+^T cells in the ascites from the combination group (**Figures 4J–L**).

Multiplex immunohistochemical analysis of intraperitoneal tumors provided spatial insights into immune remodeling. This staining confirmed a marked increase in tumor-infiltrating CD8^+^and CD4^+^T cells in the combination therapy group, corroborating the flow cytometry findings. Additionally, staining for the viral protein ICP8 indicated its predominant localization at the tumor periphery, confirming effective oncolytic infection (**Figures 5A, B**). In alignment with enhanced T-cell activation, ELISA analysis of intraperitoneal tumor homogenates demonstrated that VT1092M and combination therapy significantly increased the expression levels of IL-12 and IFN-γ compared to both the NaCl and anti-CTLA-4 antibody monotherapy groups, suggesting a robust activation of a pro-inflammatory, anti-tumor immune response (**Figure 5C**).

**Figure 5:**
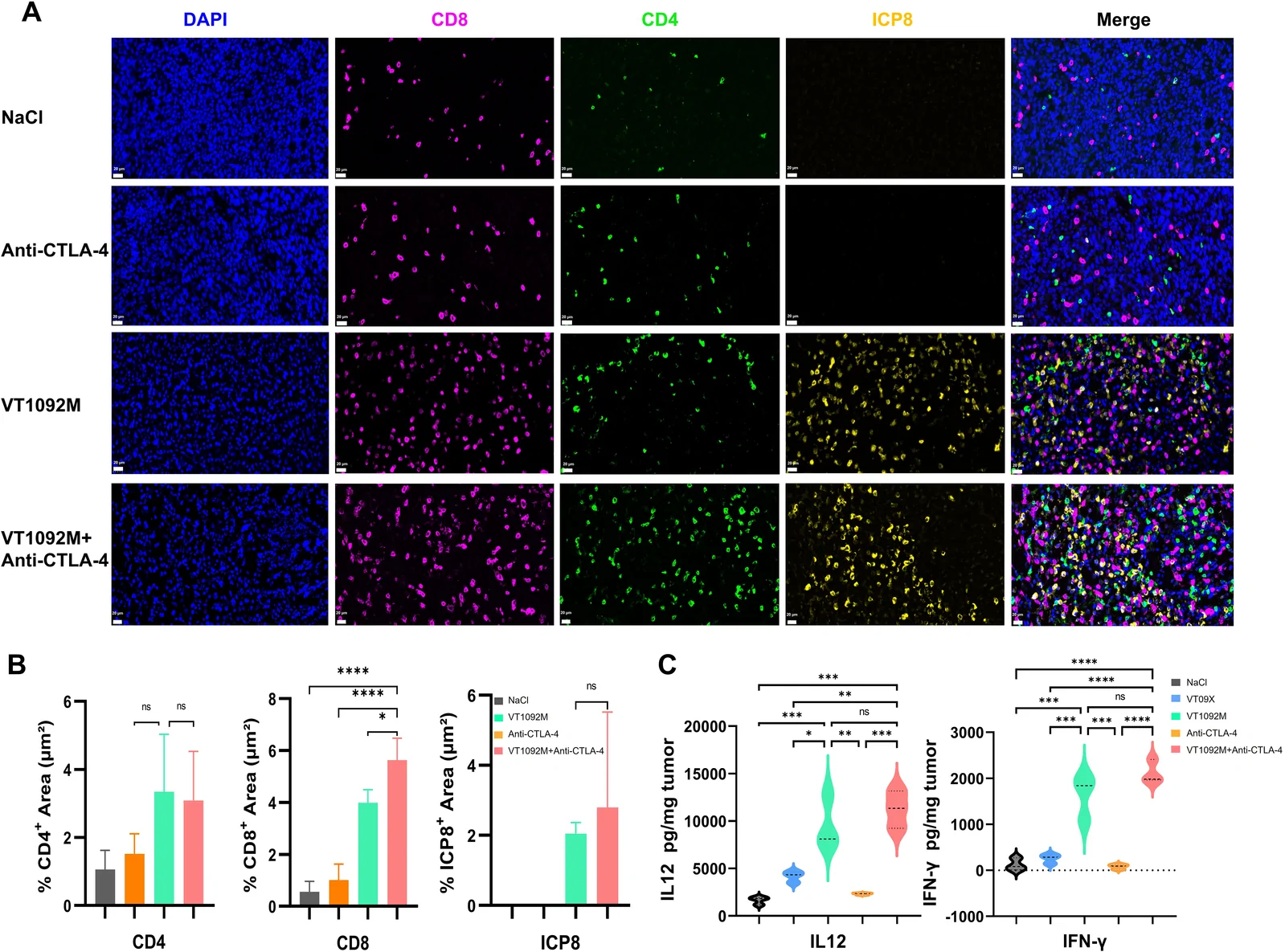
Combination therapy promotes intratumoral CD8^+^T-cell accumulation and induces expression of IFN-γ and IL-12. The intraperitoneal tumors were harvested in the NaCl, anti-CTLA-4, VT1092M, and VT1092M+anti-CTLA-4 groups after 10 days of treatment. CD8^+^ T cell was stained with CD8 (purple), CD4^+^ T cell was stained with CD4 (green), VT1092M infected cell was stained with ICP8 (yellow), and nucleus was stained with DAPI (blue). **(A)** Representative image of immunofluorescence staining of the tumor sections from mice showing infiltrated CD8^+^ T cells, CD4^+^ T cells and VT1092M infected cells. **(B)** Quantification of fluorescence intensity of CD8^+^ T cells, CD4^+^ T cells and VT1092M infected cells. **(C)** Quantitative analysis of the expression of IFN-γ and IL-12 in intraperitoneal tumors harvested in mice after 10 days of treatment by ELISA. Data are shown as mean ± SD (*n* = 3), *p < 0.05, **p < 0.01, ***p < 0.001, ****p < 0.0001.

Multiplex immunohistochemical analysis of macrophage populations demonstrated that the combination therapy significantly increased the overall proportion of macrophages (pan-macrophages, CD68^+^) and specifically enriched the M1-polarized subset (CD68^+^iNOS^+^) in comparison to both monotherapy and NaCl control groups. While the difference in M1 polarization between the combination therapy and VT1092M monotherapy did not achieve statistical significance, the incorporation of anti-CTLA-4 into VT1092M significantly decreased the proportion of M2-polarized macrophages (CD68^+^CD206^+^) relative to VT1092M monotherapy. Moreover, M1 macrophages were predominantly localized at the tumor margins, whereas M2 macrophages were more frequently observed within the tumor core. These findings indicate that combination therapy effectively activates the immune system, inducing a marked shift towards M1 macrophage polarization (**Figures 6A–F**). This shift facilitates the accumulation of these “combat-type” macrophages at the invasive tumor edges, thereby enhancing the immune response and contributing to the observed anti-tumor efficacy. In addition, staining for the vascular marker CD31 revealed significantly reduced microvessel density in the combination group compared to other monotherapies, indicating potent inhibition of tumor angiogenesis (**Figures 6G, H**). This is consistent with the induction of IL-12 expression by VT1092M infection within the tumor microenvironment, which plays a pivotal role in regulating anti-tumor immunity through the stimulation of IFN-γ secretion from T and NK cells, thereby directly inhibiting tumor angiogenesis (31–33). Furthermore, the combination of VT1092M with anti-CTLA-4 therapy is posited to enhance this effect by promoting T-cell trafficking and infiltration, which in turn increases local levels of IFN-γ and anti-angiogenic mediators, ultimately leading to superior vascular suppression in the combination treatment group (34).

**Figure 6:**
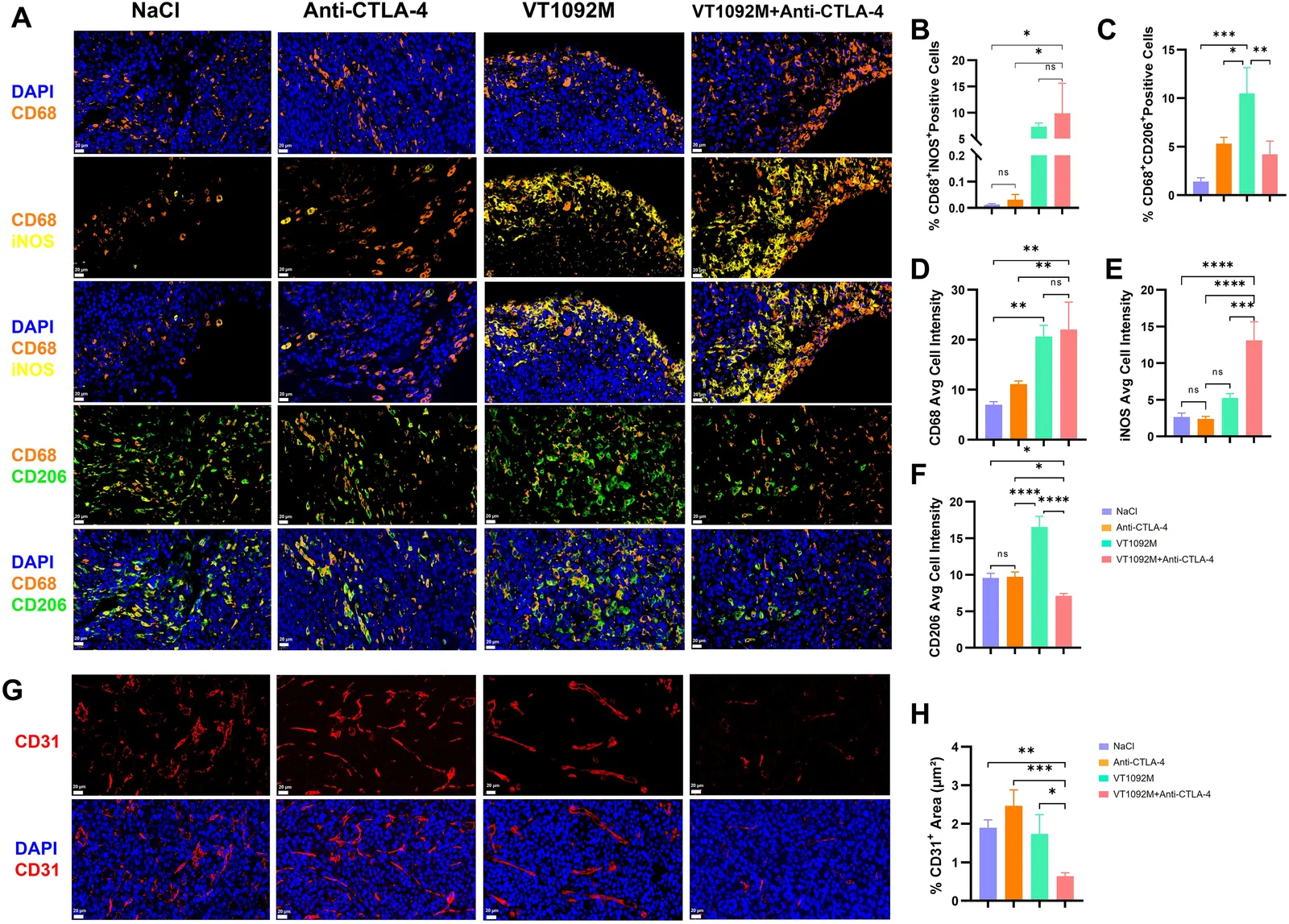
Immunofluorescence staining of M1 and M2 macrophages and CD31+ tumor vessels. Pan-macrophage was stained with CD68 (orange), M1 macrophage was stained with iNOS (yellow), M2 macrophage was stained with CD206 (green), tumor vessels were stained with CD31 (red), and nucleus was stained with DAPI (blue). **(A)** Representative images of CD68, iNOS, and CD206 staining of the intraperitoneal tumors in each group after 10 days of treatment, respectively. **(B)** Quantification of the percentage of CD68^+^iNOS^+^ cells and **(C)** CD68^+^CD206^+^ cells and immunofluorescence intensity of markers (**D–F**) in each group after 10 days of treatment, respectively. **(G)** Representative images of CD31 staining of the intraperitoneal tumors in each group after 10 days of treatment, respectively. **(H)** Quantification of the percentage of CD31^+^ area. *n* = 3, *p < 0.05, **p < 0.01, ***p < 0.001, ****p < 0.0001.

### 3.5 VT1092M combined anti-CTLA-4 therapy reprograms systemic immunity and induces tumor-specific cytotoxicity

To determine whether the VT1092M+anti-CTLA-4 combination therapy reprograms systemic immunity in mice with colorectal intraperitoneal metastases, we performed flow cytometric analysis of peripheral blood leukocytes on day 14 post-treatment (**Figure 7A**). The combination therapy induced a profound shift in the peripheral immune compartment. Specifically, it significantly increased the overall proportions of T cells and B cells compared to the NaCl, VT09X, and anti-CTLA-4 monotherapy groups, with VT1092M alone showing similar upregulation (**Figures 7B, D**). Further subset analysis revealed that the combination therapy enhanced the differentiation of CD8^+^T cells, expanding naïve, central memory, and effector memory subsets relative to other monotherapies, with a potency comparable to VT1092M alone (**Figures 7E, F**). Similarly, analysis of CD4^+^T cells showed that the combination increased the proportions of naïve and memory subsets, again paralleling the effect of VT1092M monotherapy (**Figures 7E, G**). Collectively, these data demonstrate that the combination therapy extensively reshapes the systemic immune landscape by expanding lymphocyte pools and broadening the repertoire of antigen-responsive T-cell subsets, thereby potentiating a systemic anti-tumor immune state.

**Figure 7:**
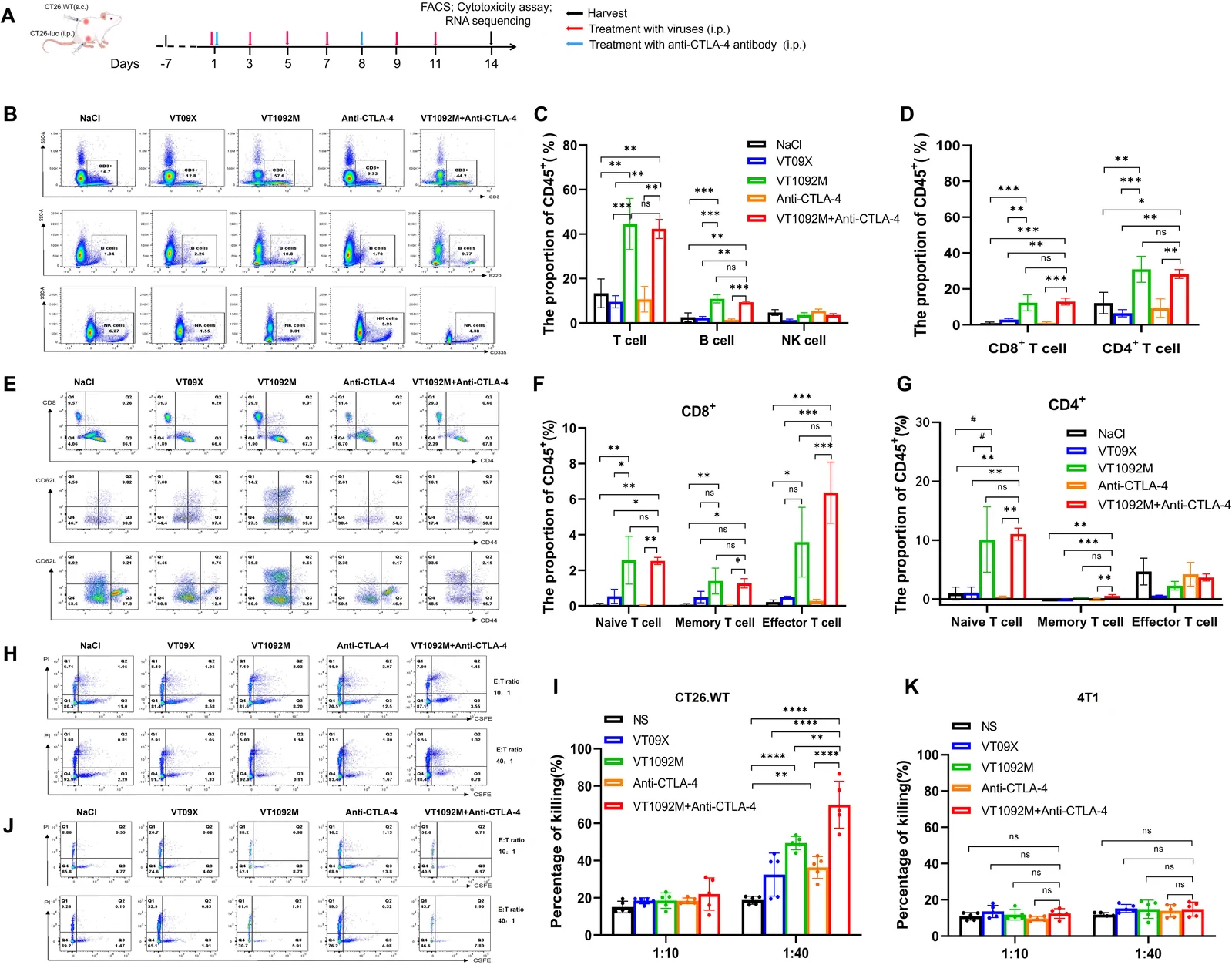
Flow cytometric analysis of the proportions of immune cells and tumor cell killing assay by CD45+ cells in the peripheral blood of mice. **(A)**The peripheral blood was collected from each group of mice two days after treatment ends. Red blood cells were lysed for immunophenotyping. **(B, C)** Representative flow diagram of T, B, and NK cells and their cell populations in the peripheral blood of mice. **(D–G)** Representative flow diagram of CD4^+^, CD8^+^ T cells and their cell populations in peripheral blood of mice. Naïve (CD62L^+^CD44^–^), central memory (CD62L^+^CD44^+^), and effector memory (CD62L^–^CD44^+^) phenotypes were defined by the expression of CD44 and CD62L. **(H)** Representative flow diagram of cytotoxic effects of CD45^+^ T cells on CT26.WT cells. The CD45^+^ cells were isolated form the peripheral blood of mice after two days after treatment ends. CT26.WT or 4T1 served as the target cells, and CD45^+^ T cells acted as effector cells (effector-target ratio = 10:1 or 40:1). **(I)** Percentage of tumor killing by CD45^+^ T cells against CT26.WT cells. **(J)** Representative flow diagram of cytotoxic effects of CD45^+^ T cells on 4T1 cells. **(K)** Percentage of tumor killing by CD45^+^ T cells against 4T1 cells (*n* = 5). The experimental results are expressed as Mean ± SD, *P < 0.05, **P < 0.01, ***P < 0.001, ****P < 0.0001.

To further evaluate tumor-specific immunoreactivity, CD45^+^ leukocytes isolated from treated mice were co-cultured with CFSE/PI-labeled CT26.WT (homologous) or 4T1 (heterologous) tumor cells. We observed that cytotoxicity against CT26.WT cells was enhanced in a dose-dependent manner, as indicated by the effector-to-target (E:T) ratio. Notably, at a 40:1 ratio, the combination of VT1092M and anti-CTLA-4 elicited significantly greater cytotoxicity compared to all monotherapy treatments (**Figures 7H, I**). In contrast, no significant cytotoxicity was observed against 4T1 cells in any treatment group (**Figures 7J, K**). These findings confirm that VT1092M+Anti-CTLA-4 selectively potentiates antigen-specific cytotoxicity against CT26.WT tumors, indicating a tumor-specific immunotherapeutic mechanism.

### 3.6 VT1092M combined anti-CTLA-4 antibody therapy reveals exploratory alterations in the tumor microenvironment transcriptome

To delineate the transcriptomic remodeling induced by VT1092M+anti-CTLA-4 combination therapy in the tumor immune microenvironment, we conducted RNA sequencing (RNA-seq) analysis on intraperitoneal and subcutaneous tumor samples (**Figure 7A**). In this exploratory analysis, the transcriptomic analysis of combination therapy revealed a distinct spatiotemporal heterogeneity in the immune response between local and distal tumors. All virus-treated groups (VT09X, VT1092M, and VT1092M+anti-CTLA-4) showed notable activation of immune-related genes in intraperitoneal tumors. In contrast, distal subcutaneous tumors exhibited a more pronounced upregulation of these genes specifically in the VT1092M and VT1092M+anti-CTLA-4 groups, indicating a trend toward more pronounced systemic immune activation. Strikingly, subcutaneous tumors from the combination group exhibited the most pronounced upregulation of key pathways, chemokines (Cxcl9/10/11, Ccl3/4/5), key effectors of T-cell activation (Cd8a, Gzmb, Ifng, Prf1, Tbx21, and Eomes), cytotoxicity (Tap1/2, B2m, and Stat1), immune checkpoints (Pdcd1), and inflammatory signaling (Il1a, Il2rg, Il15, Cd40), as well as markers associated with memory T cells, such as Il7r (CD127) and Sell (CD62L), consistent with improved immune infiltration, activation, and effector functions driven by the combination treatment (**Figure 8A**). Gene set enrichment analysis (GSEA) of the VT1092M+anti-CTLA-4 group in both subcutaneous and intraperitoneal tumors showed exploratory enrichment trends toward gene sets related to T-cell activation, inflammatory response, and apoptosis (**Figures 8B, C**). In addition, the KEGG and GO analysis indicated enrichment and alterations in pathways associated with TNF, NK cell–mediated cytotoxicity, T-cell receptor, B-cell receptor, IL-17, and PI3K signaling pathways in the combined treatment group (**Supplementary Figure S4**). This transcriptional reprogramming may suggest an enhancement in antigen recognition, immune cell trafficking, and remodeling of the pro-inflammatory tumor microenvironment.

**Figure 8:**
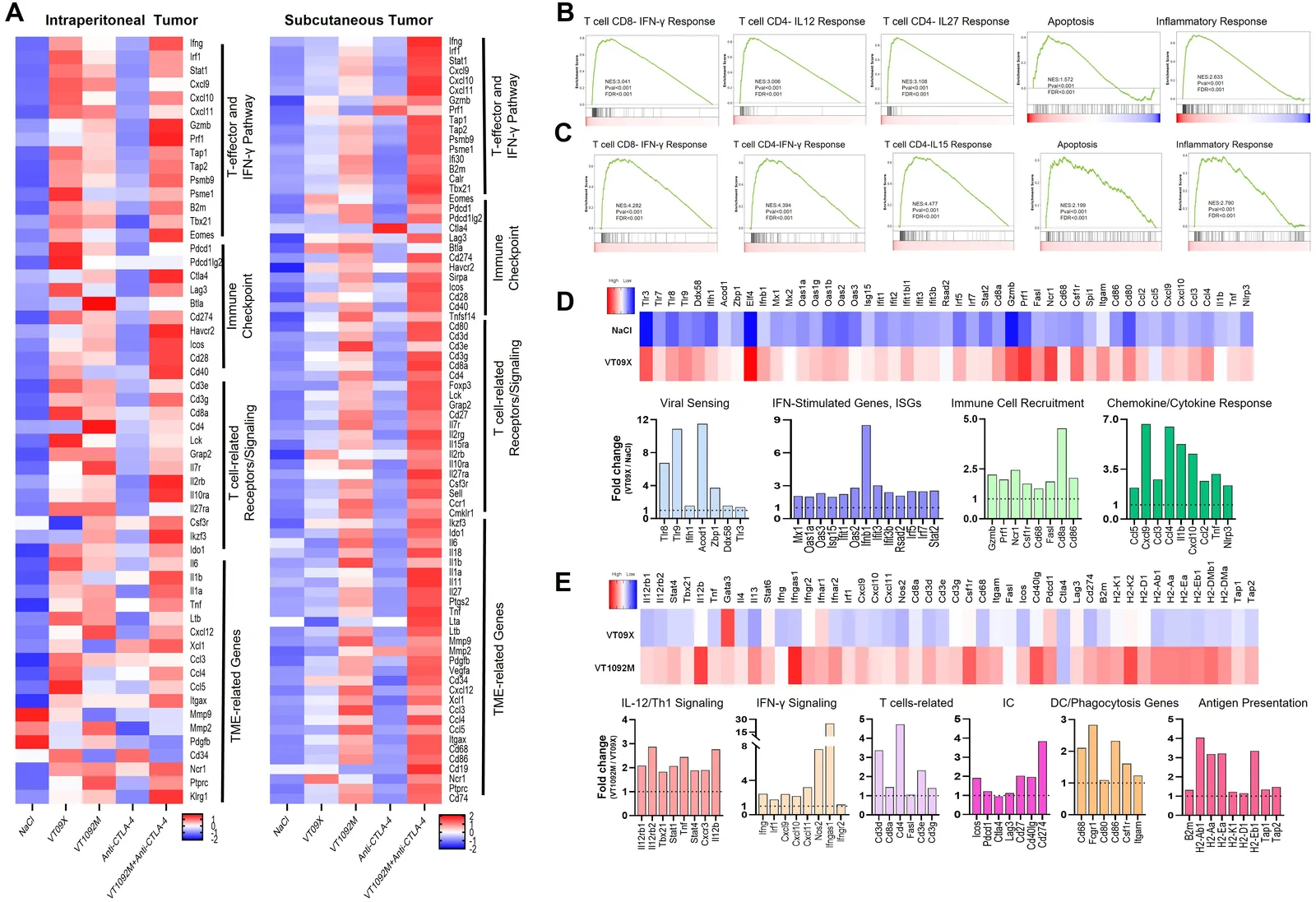
VT1092M+Anti-CTLA-4 remodeled the tumor immune microenvironment at the RNA level. The intraperitoneal and subcutaneous tumors were isolated from mice after treatment as described in **Figure 2A**. **(A)** Heat map of immune-related differential expression genes between treatments. Hierarchical clustering was performed using log_10_ (FPKM + 1) values, with red representing high expression genes and blue representing low expression genes. **(B)** GSEA of gene sets related to T cell–related pathway, apoptosis and inflammatory response in subcutaneous tumors intraperitoneal tumors **(C)** from VT1092M+Anti-CTLA-4 treated mice. **(D)** Heatmap analysis of differential gene expression related to viral infection and comparisons of gene expressions in subcutaneous tumors from the NaCl and VT09X group. **(E)** Heatmap analysis of differential gene expression related to the exogenous IL-12 pathway and comparisons of gene expressions in subcutaneous tumors from VT09X and VT1092M group.

To elucidate the distinct immunological roles of the viral backbone and the recombinant IL-12, we performed comparative transcriptomic analyses. Relative to the NaCl control, the viral backbone (VT09X) independently upregulated pathways associated with viral sensing, interferon-stimulated genes (ISGs), and immune recruitment, thereby eliciting a robust antiviral innate immune signature (**Figure 8D**). In contrast, the recombinant vector expressing IL-12 (VT1092M) was associated with notable changes in the TME, notably enhancing IL-12/Th1, interferon-gamma (IFN-γ), and T-cell functional pathways, while also upregulating genes involved in antigen presentation and immune checkpoints. These findings suggest that localized expression of IL-12 may effectively redirect virus-induced inflammation towards a Th1-dominated, cytotoxic T-cell response, potentially transforming innate immunity into a sustained adaptive antitumor response (**Figure 8E**).

## 4 Discussion

Peritoneal metastasis represents a prevalent and severe form of metastasis associated with CC, frequently characterized by substantial accumulation of malignant peritoneal ascites (5, 8). Within the peritoneal cavity, tumor cells either remain suspended in malignant ascites or adhere to surfaces, leading to immune dysfunction that diminishes the efficacy of chemotherapy and targeted therapies (31, 35). Consequently, the treatment of colorectal cancer with peritoneal metastasis presents significant challenges (36, 37). In this study, we utilized intraperitoneal administration of the OV VT1092M, which expresses IL-12 to boost anti-tumor immunity, combining direct oncolytic activity and ICI blockade as a strategy to overcome the distinctly immunosuppressive peritoneal TME and achieve durable responses in mice with intraperitoneally disseminated colorectal cancer. Our findings indicate that the OV VT1092M effectively activates the effector functions of CD8^+^ T cells, leading to the inhibition of intraperitoneal tumors and promoting tumor regression, while concurrently suppressing the formation of malignant peritoneal ascites.

ICIs have achieved notable advancements in the management of advanced cancer, however, their effectiveness in treating intraperitoneal metastatic tumors remains constrained, particularly in patients with colon cancer and intraperitoneal metastasis (38). Studies show that oncolytic virus therapy boosts T cell infiltration in the TME, enhancing cytotoxic T cell responses (22, 25–28. Immune checkpoint molecules like PD-L1 and CTLA-4 are upregulated after virus infection, increasing targets for ICIs (39). Furthermore, the combination of oncolytic viruses with ICIs can promote the accumulation of effector T cells (40–42).

In our study, monotherapy with ICIs demonstrated limited efficacy against intraperitoneal tumors and malignant ascites, indicating that our murine model exhibits resistance to immune therapy. In contrast, monotherapy with VT1092M effectively inhibited the growth of intraperitoneal tumors in murine models; however, it did not significantly impede the growth of distant subcutaneous tumors (**Figure 1E**). Conversely, the combination therapy comprising VT1092M and ICIs, specifically an anti-CTLA-4 antibody, elicited a stronger anticancer immunity that not only initiated a robust local immune clearance, resulting in tumor regression and suppression of malignant ascites formation, but also successfully induced a strong antigen-specific immune memory, thereby achieving remarkable systemic and longlasting anti-tumor effects.

Mechanistically, the combination therapy activates the anti-tumor immune microenvironment within the peritoneal cavity. Notably, there is a significant accumulation of cytotoxic CD8^+^ T cells in both the ascitic fluid and the tumor tissue, accompanied by a pronounced increase in the presence of M1 macrophages and a marked suppression of tumor-associated angiogenesis within the tumor microenvironment. The significant upregulation of IFN-γ and IL-12 within intraperitoneal tumors reveals that the therapy reprograms the local immune landscape into a pro-inflammatory, anti-tumor state. RNA-sequencing analysis indicated preliminary trends in the modulation of several tumor immune response-related pathways by the combination therapy. The viral backbone triggered a broad antiviral and inflammatory signature through viral sensing and IFN pathways, while the transgenic interleukin-12 subsequently directed this response towards a Th1-dominated, cytotoxic T-cell phenotype. Notably, the most pronounced transcriptional signature was observed in distal subcutaneous tumors, underscoring the potency of the systemically disseminated immunity, which requires validation through further experimental studies.

In the CT26 intraperitoneal metastasis model, the HSV1-IL12 OV effectively addresses the critical challenge of “immune initiation and T-cell recruitment” by transforming the immunosuppressive microenvironment into one that supports immune activity. This transformation establishes the necessary conditions for the success of anti-CTLA-4 therapy, which relies on the presence of pre-existing T cells. However, the efficacy of HSV1-IL12 monotherapy is limited by the inadequate strength and scope of the immune response; the activated T cells are susceptible to exhaustion or suppression and do not sustain systemic T-cell functions over time. The integration of HSV1-IL12 with anti-CTLA-4 therapy is essential for achieving sustained distal effects and preventing tumor recurrence. Interleukin-12 (IL-12) is instrumental in the induction of central memory T cells (Tcm), while CTLA-4 blockade promotes the differentiation of effector T cells into memory T cells, thereby preserving their functionality. This combined therapeutic strategy enhances the generation of a greater number of tumor-specific memory T cells with improved durability and function. These memory T cells circulate throughout the body, enabling a rapid response to tumor cells at any location, including distant and newly emerging tumors, thereby ensuring prolonged and systemic immune surveillance. The combination of HSV1-IL12 and anti-CTLA-4 modifies the local microenvironment, significantly activates high-quality T cells, inhibits suppressive signals, establishes robust immune memory, and empowers the systemic immune system to effectively target distant lesions. Additionally, the dual subcutaneous and intraperitoneal tumor model used in this study is practical for studying peritoneal and systemic immunity, but its limitations, especially the immunological differences between primary and metastatic tumors, should be considered when applying findings to clinical peritoneal metastasis.

## 5 Conclusions

In summary, intraperitoneal injection of OV VT1092M with ICIs, especially anti-CTLA-4 antibody, cannot only significantly enhance suppression of intraperitoneal metastatic cancer but also elicit durable tumor-specific immune memory and prolong survival of mice, showing great potential in clinical application for the treatment of intraperitoneal metastasis of CC.

## Data Availability

The original contributions presented in the study are included in the article/**Supplementary Material**. Further inquiries can be directed to the corresponding author.
