# Supplementary material for: Oncolytic virus VT1092M synergizes with immune checkpoint inhibitors against intraperitoneal metastases of colorectal cancer
Source: Front Immunol. 2026 Sep 30;17:1926207. doi: 10.3389/fimmu.2026.1926207 (PMC13662124; doi:10.3389/fimmu.2026.1926207)
Supplement: Supplementary file 1 [file Table1.docx]

**Supplementary Information**

**Oncolytic Virus VT1092M Synergizes with Immune Checkpoint Inhibitors against Intraperitoneal Metastases of Colorectal Cancer**

**Authors:** Zongfeng Hu^1,a^, Xinru Yuan^1,a^, Shuzhen Li^1,a^, Wenqi Liu^a^, Jiajia Liu^b^, Hua Zhou^b^, Chunyang Sun^b^, ChaoTian^b^, Kaixuan Xue^a^, Yiting Cheng^a^, Jinyu Wang^a^, Haitian Xu^a^, Jianshuai Yang^b^, Xiaopeng Li ^a, b,^*, Jingfeng Li ^a,^*

*Corresponding author. E-mail: JL: [jfli@genevec.com](mailto:jfli@genevec.com), XL: [patricklee@genevec.com](mailto:patricklee@genevec.com)

**Author details**

^a^ School of Pharmacy, Yantai University, Yantai 264005, Shandong, China

^b^ Beijing WellGene Company, Ltd, Beijing 100085, China

^1^ These authors contributed equally to this work.

**Keywords:** Oncolytic virus; HSV-1; ICIs; intraperitoneal metastasis; colon cancer

**Table S1.** Primers for qPCR analysis.

| Primer-F | CCAATCCATACTCAGCGTAACG |
| --- | --- |
| Primer-R | GTTCCGACTCCGTGGATCT |
| probe | 5’-FAM-CAGCCCCTGGCGAGA-MGB-3’ |

**Figure S1**. qPCR analysis of biodistribution of the oncolytic virus in mice tumors and major organs following intraperitoneal administration. Not Detected:ND; Below the lower limit of detection : LLOD.

**Figure S2**. (A) Representative images of the parietal peritoneum and its blood vessels in the indicated groups on days 6 and 10 after administration. (B) Representative

images of malignant ascites and (C) statistical comparison of ascites volume in the indicated groups on days 6 and 10 after administration.

**Figure S3**. The gating strategy of flow cytometric analysis of T, NK, B cells in abdominal tumors and ascites from the indicated groups. Single-cell suspensions from mice were first gated on live CD45^+^ single cells before gating on CD3^+^ cells or CD3^-^ cells. Lin markers were CD3, CD8, CD4, CD62L, CD44, CD335, and B220. FSC-A, forward scatter area; SSC-A, side scatter area.

**Figure S4**. (A–B) KEGG pathway analysis results for DEGs in subcutaneous tumors from VT1092M+Anti-CTLA-4 group compared to VT1092M or Anti-CTLA-4 group. (C-D) GO Top 30 term analysis results for DEGs in subcutaneous tumors from VT1092M+Anti-CTLA-4 group compared to VT1092M or Anti-CTLA-4 group.
